# An unusual flight-associated occipital headache

**DOI:** 10.17712/nsj.2017.1.20150511

**Published:** 2017-01

**Authors:** Shivika Nath, Ashok K. Saxena

**Affiliations:** *From the Pain Clinic, Department of Anesthesiology and Pain Medicine, College of Medical Sciences, Guru Teg Bahadur Hospital, University of Delhi, Delhi, India*

In the medical literature, various types of headaches have been categorized and reported but the headache associated with airplane travel which develops during landing is a new entity. Recently it has been categorized in international headache society classification.^1^ It is different from other types of headaches since here neurological and otolaryngological examinations as well as imaging are normal. We hereby report 2 male patients (aged 49 years and 42 years) with history of severe, sharp shooting, stabbing, piercing headache in bilateral occipital and parieto-occipital region associated with the descent of airplane and landing.

They presented to us in the pain clinic with intense bilateral occipital and parieto-occipital severe sharp shooting stabbing and piercing headache associated with severe dizziness every time they travelled in airplane (3 times in last 7 months) and occurred during airplane descent which resolved at 40-50 minutes after landing till they arrived to conveyor belt. During each travel the headache was almost similar in intensity. The pain was also associated with mild conjunctival injection and watering of eye. First patient categorized pain score of 9/10 on numeric rating scale and second patient at 8/10 on NRS. The headache in both the patients was not associated with nausea, vomiting, visual problems, photophobia, phonophobia or osmophobia and any neurological and anatomical manifestations. Refraction testing in both cases was normal and there was no evidence of raised ICT. No history of fever was there nor was diurnal variation of headache elicited. There was no past history of tension headache, cluster headache, migraine, sinusitis, facial pain squeezing and diabetes mellitus, hypertension, ischemic heart disease, stroke, convulsions, transient ischemic attack. His detailed neurological, otolaryngological and ophthalmological examination was normal and all blood tests were also within normal limits. The possibility of aerosinusitis (barotraumas or sinus squeeze) was ruled out in these patients by the Ear, nose, & throat (ENT) specialist and also by CT & MRI of paranasal sinuses. In family history, the elder sister of 49 year old male patient had a history of migraine for 5 years, no other history was positive. In both the patients, there was no history of fasting, and before the air travel, they had regular meals as well as snacks and tea inside flight. They did not have alcohol and had plenty of water and were walking around plane at regular intervals.

Based on their history and examination, as a matter of guidance for future air travel, both the patients were advised to take prophylactic oral medication in the form of tab Rizatriptan 10 mg and tablet Ultracet (Tramadol, New Delhi, India, plus Acetaminophen, New Delhi, India, 325 mg) at 45 minute (min) before the scheduled descent of airplane and depending on duration of flight. However this will always remain a matter of debate that whether triptans should be advised prophylactically in such a category of patients. They were further instructed for follow up visits subsequently.

The present case report of 2 patients highlights the possibility of precipitation of an episode of acute attack of severe headache following descent and landing of flight. It is a well-established fact that during flight travel various things can happen that shall predispose and make a passenger’s head scuffled. Also, recently several cases of airplane headaches have been published.^2^-^4^ In 2007, Mainardi F et al,^2^ in an interesting case report of a patient who presented with severe bursting headache associated to airplane travel, formalized a list of provisional diagnostic criterion for the symptoms entity and proposed for the inclusions of this type of headache in International classification headache disorders. Later on in 2012 Mainardi F et al,^5^ in an online publication carried out a survey of 75 patients suffering from airplane headache with a questionnaire based on previously proposed diagnostic criterion and was proved to be valid when applied to large number of patients suffering from this condition. He also supported for the inclusion of this type of headache in International classification of Headache disorders (ICHD) and they classified it in a distinct category in the third edition of ICHD.^1^

On the other hand, considering the diagnostic Characteristic by Mainardi F,^5^ present clinical reports of both the patients were partially fulfilling the above criteria, including severity, also they do not have any positive medical history or past history and family history, but the site of pain in our cases were bilateral occipital and parieto-occipital where as it was frontal and peri-orbital in the above criterion, and the duration of pain lasted for 40-50 min, however it was less than 20 min in the diagnostic criterion.

It has been reported that most of the airplane headache patient suffer from other form of headache (migraine and tension type headache) without any causative role.^3^ However in our patients they had no past history of migraine, tension headache, cluster headache and MRI of the brain was absolutely normal. Regarding the age, several articles have been published reporting the occurrence of airplane headache in younger individuals. However in our case age of both patients were between 40-50 years which has been reported in few publications, such as in a case series by Mainardi et al^2^ and in one case report by Berilgen^5^ in patients of airplane headache.

Till now various pathophysiological mechanisms for the airplane headache have been proposed; recently Berilgan et al^4^ described barotraumas like mechanism, primarily related to rapid change in atmospheric pressure, and it mainly manifest during landing of airplane. “Barotraumas caused by pressure changes during take off and landing affect ethmoidal nerve (branch from the ophthalmic branch of the trigeminal nerve) that carries sensory innervations and the mucosa on the inner surface of the paranasal sinuses or the nociceptions in ethmoidal arteries thus activating the trigeminal vascular system and leading to headache in periorbital and retroorbital area”. However the headache in occipital and parieto-occipital area in our patients is still unexplained. The airplane headache explained above is very different from high altitude headache which develops within 24 hours of ascent and associated with Cheyenne-Stokes respiration and classified in distinct category in international headache society.

In the past, some authors described that on ascent, the air in the paranasal sinuses obviously will expand as based on Boyle’s law and contract during descent of aircraft. The phenomenon of squeeze happens on descent, when air trapped in the sinuses, contract and predispose to development of pressure gradient, hence air is ultimately shifted to center of sinuses, predisposing to mucosal edema and or submucosal hematoma, predisposing to further occlusion of sinus atrium and thus aerosinusitis develops. But in our patients no evidence of aerosinusitis is present as ruled out by ENT examination and CT paranasal sinuses. Regarding the prophylactic management of airplane headache in our patients, we advised for medication, which is to be appropriately timed and titrated so that depending on the duration of air travel towards its destination, the peak action of this prophylactic medication should be able to prevent any further episode of flight-associated headache.

We conclude that although the exact pathogenesis of headache attributed to airplane travel remains unclear the interaction between anatomical, environmental and local aspects leading to multifunctional mechanism should be advanced. Also there is an urgent need for detail clinical workup regarding pathophysiological causes of the same. An awareness program needs to be taken up with all airlines across the globe informing about this type of headache. This shall help in getting vital inputs from the passengers about the exact incidence of flight headache thus helping in better management of such headaches. All such inputs shall become source of basic information and pave way for further therapeutic researches and strategies thus reducing incidence of flight headaches. It is always a possibility, unless until ruled out that genetic makeup of a patient may predispose to fight associated headache. At present the passengers should be made aware about this peculiar headache, so that they can take the appropriate medical advice and prophylactic medication before any planned air travel.
